# Colorimetric Sensing with Gold Nanoparticles on Electrowetting-Based Digital Microfluidics

**DOI:** 10.3390/mi12111423

**Published:** 2021-11-19

**Authors:** Zhen Gu, Jing-Jing Luo, Le-Wei Ding, Bing-Yong Yan, Jia-Le Zhou, Jun-Gang Wang, Hui-Feng Wang, Cong Kong

**Affiliations:** 1 Key Laboratory of Advanced Control and Optimization for Chemical Processes Ministry of Education, East China University of Science and Technology, Shanghai 200237, China; guzhen@ecust.edu.cn (Z.G.); ecust_ljj@163.com (J.-J.L.); 10182191@mail.ecust.edu.cn (L.-W.D.); zhou.jiale@ecust.edu.cn (J.-L.Z.); 2 School of Chemical and Environmental Engineering, Shanghai Institute of Technology, 100 Haiquan Road, Shanghai 201418, China; jgwang@sit.edu.cn; 3 Shanghai Key Laboratory of Forensic Medicine, Academy of Forensic Science, Shanghai 200063, China; 4 Key Laboratory of East China Sea Fishery Resources Exploitation, Ministry of Agriculture and Rural Affairs, East China Sea Fisheries Research Institute, Chinese Academy of Fishery Sciences, Shanghai 200090, China

**Keywords:** digital microfluidic, gold nanoparticle, electrowetting, colorimetric sensing, analytical instrument

## Abstract

Digital microfluidic (DMF) has been a unique tool for manipulating micro-droplets with high flexibility and accuracy. To extend the application of DMF for automatic and in-site detection, it is promising to introduce colorimetric sensing based on gold nanoparticles (AuNPs), which have advantages including high sensitivity, label-free, biocompatibility, and easy surface modification. However, there is still a lack of studies for investigating the movement and stability of AuNPs for in-site detection on the electrowetting-based digital microfluidics. Herein, to demonstrate the ability of DMF for colorimetric sensing with AuNPs, we investigated the electrowetting property of the AuNPs droplets on the hydrophobic interface of the DMF chip and examined the stability of the AuNPs on DMF as well as the influence of evaporation to the colorimetric sensing. As a result, we found that the electrowetting of AuNPs fits to a modified Young–Lippmann equation, which suggests that a higher voltage is required to actuate AuNPs droplets compared with actuating water droplets. Moreover, the stability of AuNPs was maintained during the processing of electrowetting. We also proved that the evaporation of droplets has a limited influence on the detections that last several minutes. Finally, a model experiment for the detection of Hg^2+^ was carried out with similar results to the detections in bulk solution. The proposed method can be further extended to a wide range of AuNPs-based detection for label-free, automatic, and low-cost detection of small molecules, biomarkers, and metal ions.

## 1. Introduction

In the last decades, digital microfluidics (DMF) have attracted widespread attention as a new lab-on-a-chip approach for programmable manipulating of droplets at the volume of nano-to-micro liters [1,2]. The droplets on the DMF chip can be actuated in parallel to mix, separate, and dispose, which can be used in wide applications such as chemical reactions, automatic detection, and cell culture [3,4,5]. DMF based on electrowetting on dielectric (EWOD) is promising to be used in portable applications such as deployable environmental monitoring and point-of-care testing (POCT) [6], since it drives the droplets only requiring configuration of the voltage applied on an electrode array, which has advantages including accurate volume control, small size, low reagent cost, and low power consumption [7,8,9,10]. The key to actuating the droplet on DMF is to fabricate a dielectric layer on the electrode array with features such as thin, insulated, and hydrophobic [11]. Then, a lateral force on the droplets will be generated by applying a biased voltage between the electrodes. The direction and velocity of droplet movements can be programmed according to the detection process even after the fabrication of the EWOD-based DMF chips. As various methods have been developed to fabricate DMF chips based on substrates such as silicon wafer, ITO glass, PCB board, and flexible polymers, the DMF technique is able to combine with a variety of analytical methods such as electrochemiluminescence [12], electrochemistry [13], and colorimetric sensing [14]. Currently, the EWOD-based DMF has been widely used in biosensing, environmental monitoring, and food safety, such as single-cell sequencing [2,15], cell invasion monitoring [5], detection of growth factors in embryo culture medium [9], bacterial classification [16], marine pollution monitoring [17], and detection of food nitrite [14].

Nanomaterials have been widely used in analytical methods as sensors or indicators. Among these, gold nanoparticles (AuNPs) with unique optical and chemical properties are widely used for the detection of small molecules, heavy metals, and biological molecules, including DNA, RNA, and proteins [18]. The colorimetric assay based on AuNPs has the advantage of low limit of detection (LOD), because the interaction between the surface electrons on the AuNPs and the incident light causes the effect of localized surface plasmon resonance (LSPR), which highly amplify the light scattering of AuNPs [19]. Usually, colorimetric techniques based on AuNPs is realized by modulating the distances between AuNPs with target molecules. For example, the trans-cleavage activity of CRISPR/Cas12a is used to facilitate the assembly of AuNPs for the detection of SARS-CoV-2 [20]. Antibiotics are detected based on its adsorption on the AuNPs, which prevents the color changes induced by the aggregation of AuNPs by the addition of salt solution [21]. Therefore, the AuNPs is promising to be applied as sensors for automatic detections based on the DMFs. Although previous studies have demonstrated the possibility to control nanosuspensions by using electrowetting [22], the electrowetting property of AuNPs and the effect of solvent evaporation to the results of colorimetric sensing on DMFs were not concerned.

With these insights, in this study, we developed a novel DMF system for the manipulation of AuNPs droplets based on electrowetting and examined the ability of AuNPs for colorimetric sensing on the DMFs. We first measured the mobility of AuNPs droplets on the DMF chip. Then, the stability of AuNPs droplets during electrowetting was also investigated by monitoring the droplet absorbance during actuating on the DMF chip. In addition, the evaporation effect to the colorimetric sensing was also evaluated. As a module experiment, the detection of Hg^2+^ based on AuNPs was automatically conducted by using the proposed DMFs with integrated two-wavelength measuring module, and the results were consistent with the detections performed in a bulk solution, which suggests that the proposed method could be further developed for a versatile analytical platform for portable applications, such as point-of-care testing, in-field environment monitoring, and food safety.

## 2. Materials and Methods

### 2.1. Materials and Reagents

Hg^2+^ standard solution (100 mg/L) was purchased from Inorganic Ventures, Inc., USA. Trisodium citrate, lysine, HAuCl_4_ was purchased from Sinopharm Chemical Reagent Co., Ltd. All buffers and solutions were made with deionized water purified by Milli-Q (Millipore, Billerica, MA, USA).

### 2.2. Setup of DMF Platform

**Figure 1:**
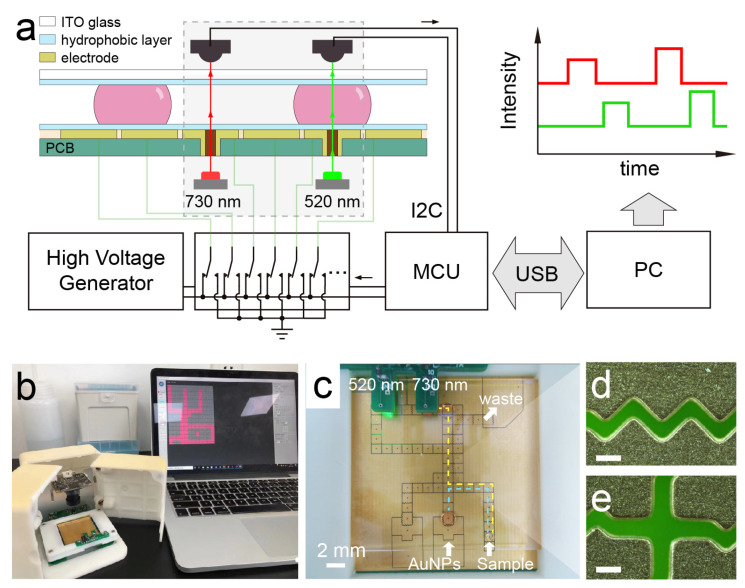
(**a**) Scheme of the digital microfluidic system for colorimetric detection at two wavelengths (730 nm and 520 nm). (**b**) Photograph of the digital microfluidic system. (**c**) Photograph of the digital microfluidic chip integrated with optical sensing elements. (**d**,**e**) are the microscopy images of the cross-fingers of the electrodes and the gaps between the electrodes. The scale bar equals 100 μm.

An integrated DMF system was used for the automatic manipulation and sensing of the droplets as shown in Figure 1, which contains a two-plate DMF chip, optical sensor (TSL2571, ams AG, Graz, Austria), LED light source (520 nm, XLamp, Cree Inc., Durham United States), and control board with a microcontroller [23]. The digital camera is vertically mounted above the DMF chip. As shown in Figure 1a, the bottom plate of the DMF chip was made of printed circuit board techniques (FASTPCB Technology, Shenzhen China), which comprised 58 actuation electrodes (2.25 mm × 2.25 mm) and 3 reservoir electrodes (9.72 mm × 6.95 mm) with 0.1 mm gaps between each. The edges of the electrodes were designed with cross-fingers to promote the movement of droplets (Figure 1c–e). A PTFE film (10 μm, Hongfu Material, Dongguan, China) tightly attached on the electrode region of the bottom plate performs as both dielectric and hydrophobic layers. The top plate was made of indium tin oxide (ITO) glass with a spin coating of CYTOP (CTX-809SP2, AGC Inc., Tokyo, Japan) for surface hydrophobization. To increase the sensitivity of colorimetric sensing, the DMF chip was assembled by stacking the two plates with a gap of 0.9 mm by using double-sided tape as spacers. The control board was used for executing the movement of droplets, acquiring data from the optical sensor and communicating with computer software. A 3D-printed case was designed for the whole system to reduce the influence of environmental light on optical sensing.

### 2.3. Synthesis and Characterization of AuNPs

AuNPs were prepared by the reduction of HAuCl_4_ with trisodium citrate according to the reported procedure as follows [18]: 0.1% trisodium citrate was rapidly added to a boiling aqueous solution of 0.01% HAuCl_4_. After the solution was heated for 20 min with vigorous stirring, the wine red AuNPs were obtained. Then, the products were cooled at room temperature. The average particle size of the AuNPs was determined to be 23.1 ± 4.5 nm from SEM. The concentration of the AuNPs (0.25 nM) was evaluated based on the size and its absorbance at 450 nm measured by a UV/Vis spectrometer [24]. The contact angle of the AuNPs droplets controlled by EWOD was measured through a contact angle meter.

### 2.4. Absorbance Measurement of Droplets

To measure the absorbance of the droplets at both 520 nm and 730 nm on the DMF, two apertures were fabricated in the sensing electrodes as light path (Figure 1). A two-wavelength measuring module with two LEDs (520 nm/730 nm) and two light-to-digital converters were integrated in the system. The LEDs were used as a light source and mounted below the apertures, respectively. Light-to-digital converters (TSL2571, ams AG, Graz, Austria) were mounted above the apertures for continuously acquiring the light intensity and transmitting the data to a microcontroller by using the I2C protocol. The absorbance of AuNPs was measured when the droplets moved to the sensing electrode. 

## 3. Results and Discussion

### 3.1. Manipulation of the AuNPs Droplets on DMF

**Figure 2:**
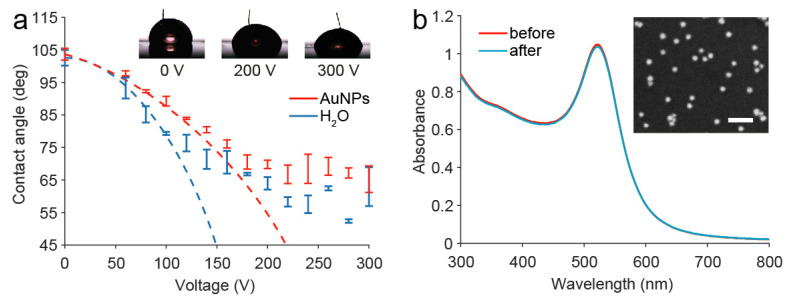
(**a**) Contact angles of the AuNPs droplets and water droplets under the applied voltage. Each data point is the mean of three measurements and error bars show standard deviation. (**b**) UV/Vis absorbance spectra of the AuNPs before and after the experiment and the SEM image of the synthesized AuNPs (inset, the scale bar equals 100 nm).

For EWOD-based DMF, the lateral force to drive the droplets comes from the changing of contact angle by applying an electric field, which usually follows the Young–Lippmann equation (Equation (1)). As the AuNPs are dispersed in aqueous solution, it is reasonable to be controlled by the electrowetting DMF. Meanwhile, the interactions between nanoparticles and the solvent under high electric field may not be ignored. To evaluate the significance of the electrowetting effect on the AuNPs droplets, the changing of the contact angle of both the AuNPs droplets and water droplets with a volume of 10 μL on the DMF chip was measured by applying a DC voltage ranging from 0 to 300 V. Here, AuNPs with a diameter of ≈25 nm was studied, as it is widely used in colorimetric assays. As a result, the contact angles of both the droplets were almost the same (≈105°) without applied voltage (Figure 2a). With the increase in applied voltage, the contact angle of water droplets appears to be lower than that of AuNPs droplets before the saturation of contact angle. The results of the water droplets before contact angle saturation were fitted to the Young–Lippmann equation (R^2^ = 0.92). On the contrary, the results of the AuNPs droplets were not fitted to the Young–Lippmann equation, which states that the EWOD force is not proportional to the square of the applied voltage. The difference in the electrowetting contact angle between the AuNPs droplets and the water droplets may result from the change in interfacial energy induced by the interaction of nanoparticles at the solid–liquid interface of the AuNPs droplets [22,25]. Therefore, a modified form of the Young–Lippmann equation for nanosuspensions by adding an item representing the change of the solid–liquid surface tension was used to fit the electrowetting contact angle of AuNPs droplets [22]. As shown in Equation (2), the effect of the AuNPs increases linearly with the applied voltage, where *A* is a constant, and *χ* is the effect of nanoparticle concentration/volume in one lateral dimension. The fitting result was agreed well with the experimental data (R^2^ = 0.99), supporting that the modified form of the Young–Lippmann equation was suitable to explain the electrowetting of the AuNPs droplets. Therefore, the result suggested that a higher applied voltage is needed to actuate the AuNPs droplet than for the water droplet to ensure the success of the manipulation, which should be taken into consideration in practice.

(1) $$\cos\theta =\cos\theta_{0}+\frac{\varepsilon_{r}\varepsilon_{0}}{2\gamma_{LG}d}V^{2}$$

(2) $$\cos\theta =\cos\theta_{0}+\frac{\varepsilon_{r}\varepsilon_{0}}{2\gamma_{LG}d}V^{2}+A\chi^{1/3}V$$

To examine the stability of the AuNPs actuated by the DMF, the AuNPs droplets were manipulated to be moving for 5 min (≈600 mm moving distance), and its UV/Vis absorption spectra were measured before and after the movement (Figure 2b). The results indicated that there was no aggregation or sediment in the AuNPs droplets as no additional peak was observed in the UV/Vis absorption spectra and the absorbance spectra of the AuNPs was nearly equal before and after the experiment. This reveals that the AuNPs is stable to be controlled by the DMF. Therefore, it could be further used in applications for DMF-based automatic colorimetric detections.

### 3.2. Evaporation Effect during the Colorimetric Sensing of AuNPs

**Figure 3:**
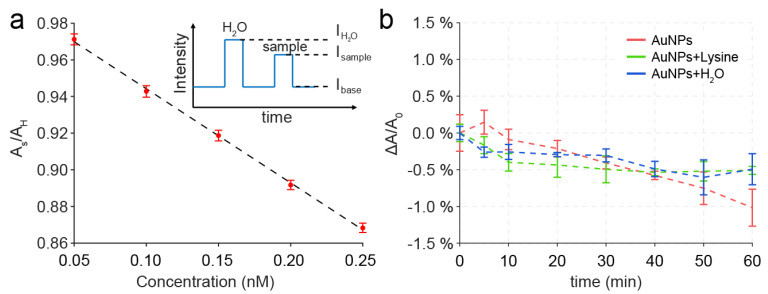
(**a**) The absorbance of droplets is linear to the concentration of the AuNPs (N = 3); Inset: diagram for the evaluation of the related absorbance, A_H_ = I_H2O_/I_base_, A_s_ = I_sample_/I_base_. (**b**) Changing of the absorbance of AuNPs droplets (5 μL) induced by the evaporation on the DMF chip, where ΔA/A_0_ = (A_s_/A_H_)/(A_s,0_/A_H,0_) − 1 is the change of absorbance as a related error to the baseline at 0 min.

The evaporation effect of the AuNPs droplets on the DMF was studied to examine if the evaporation of solvent in the droplet could influence the result of the sensing based on AuNPs. Before this, we first measured the absorbance of the AuNPs droplets with concentrations from 0.25 to 0.05 nM on the DMF. During the experiment, when the droplets moved into the sensing electrode, the light intensity would abruptly increase because of the focusing effect of the droplet (Figure 1a). As the evaporation will increase the concentration of AuNPs in the droplets, the absorbance at approximately 520 nm of the droplets was measured to quantify the evaporation, which is linear to the concentration of the AuNPs (Figure 3a). Since the concentration of a water droplet (A_H_ = I_H20_/I_base_) should not change during evaporation, it was used as a reference to eliminate the drift of optical sensor. This method could be useful to investigate the droplet evaporation on the DMF chip, as it provides the dynamic information of the droplets. As shown in Figure 3b, the ΔA/A_0_ of the AuNPs droplets with a volume of 5 μL slightly decreased with time, where ΔA/A_0_ = (A_s_/A_H_)/(A_s,0_/A_H,0_) − 1 is the change of absorbance as a related error to the baseline at 0 min. The decrease in ΔA/A_0_ was less than 0.5% within 10 min, revealing that the evaporation can hardly affect the measurement if the assay lasts several minutes. In a previous study, a complicated process was observed during the evaporation of droplets in an open configuration on a hydrophobic surface [26]. As the height of the droplet changes during evaporation, it has a significant effect on the absorbance. In this study, a two-plate configuration of DMF was used for droplet control. Therefore, the height of the droplet was constant until most of the droplet solvent was dried up, and the concentration of the AuNPs plays the main role in the measurement during evaporation. On the other hand, for detections that endure for longer time, it is feasible to fill the DMF chip with silicon oil to prevent the evaporation of droplets, which can be easily realized in a well-sealed DMF chip [27].

### 3.3. On-Chip Detection of Hg^2+^

**Figure 4:**
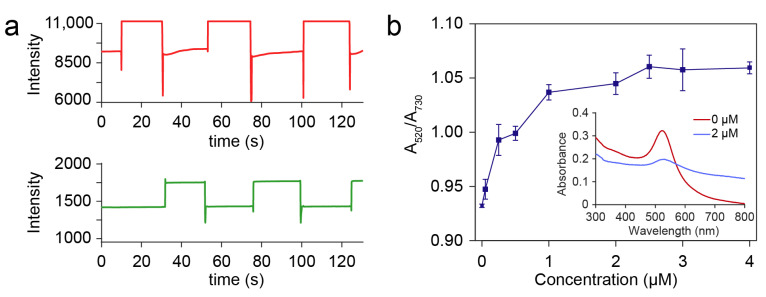
(**a**) Raw data of the absorbance with Hg^2+^ concentration of 2 μM at 730 nm (Red) and 520 nm (Green). (**b**) A_520_/A_730_ ratio of the AuNPs droplets reacted with Hg^2+^ concentration ranging from 0, 0.05, 0.25, 0.50, 1.00, 2.00, 2.50, 3.00 to 4 μM (inset: the UV/vis absorbance spectra with Hg^2+^ concentration of 0 μM and 2 μM).

To validate the sensing strategy, a proof-of-principle experiment was conducted by integrating the DMF system with a two-wavelength measuring module. The DMF system was used to automatically detect the concentration of Hg^2+^ based on AuNPs (Figure 1c). The AuNPs mixed with lysine were used as a reagent to detect and quantify the concentration of Hg^2+^. The principle of the detection is referred to a two-step mechanism by promoting the response of AuNPs to the Hg^2+^ with lysine as previously reported [18], which also has the advantage of high selectivity: First, the Hg^2+^ in the droplet was reduced on the surface of AuNPs to form a shell. Then, the AuNPs covered with Hg^2+^ were aggregated, which was induced by the interaction between lysine and the shell of Hg, resulting in a rapid color change from red to purple due to the effect of the LSPR [19]. During the experiment, sample droplets with different concentrations ranging from 0, 0.05, 0.25, 0.50, 1.00, 2.00, 2.50, 3.00 to 4 μM were loaded on the DMF chip with a volume of 2 μL. The reagent droplet (5 μL of AuNPs buffer and 1 μL of 1 mM lysine) was loaded on the DMF and mixed with the sample droplet. Then, a process for promoting the reaction was conducted by moving the droplets around the chip for 3 min. Mixed droplets were driven to the sensing electrodes for measuring the absorbance at 520 nm and 730 nm (Figure 4a). We define the absorbance ratio as A_520_/A_730_, where A_520_ and A_730_ were the related absorbance (A_s_/A_H_) at 520 nm and 730 nm, respectively. The change of the absorbance ratio of the AuNPs (A_520_/A_730_) has a good relation with the concentration of the Hg^2+^ (Figure 4b), which is consistent with the results, as previously reported in bulk solution [18]. A similar trend was observed in the UV/vis absorbance spectra of the mixed droplets with Hg^2+^ concentrations of 0 μM and 2 μM by both measuring a collection of 200 μL mixed droplets. In addition, the limit of detection based on a signal-to-noise ratio of 3:1 was only 0.01 μM, which is lower than the limit of acceptable Hg^2+^ concentration in drinkable water (0.03 μM) according to the WHO. With the increase in the Hg^2+^ concentration, the A_520_/A_730_ was almost saturated. This is reasonable, since most of the AuNPs would be aggregated at an excessive concentration of Hg^2+^ [20]. The dynamic range can be further optimized by adjusting the concentration of AuNPs, the volume ratio of the AuNPs droplet and the sample droplet, and the concentration of lysine [28].

## 4. Conclusions

In summary, we studied the EWOD property of the AuNPs droplets on the DMP chip, which reveals that the interaction of the AuNPs at the solid–liquid interface of a droplet could influence the electrowetting force for the droplet manipulation. The electrowetting contact angle of the AuNPs follows a modified Young–Lippmann equation with an additional item representing the changing of solid–liquid surface tension. Therefore, a higher voltage is needed to manipulate the AuNPs droplets than the water droplets at the same condition. We also found that the evaporation of the solvent in the AuNPs droplets had a limited influence (<0.5%) on the colorimetric sensing within 10 min. As an example experiment, we conducted the AuNPs-based colorimetric sensing of the concentration of Hg^2+^ on the DMF platform. The sensitivity of the detection was modified by introducing a two-wavelength measuring method. The results indicated that the concentration of Hg^2+^ could be determined by using the AuNPs-based method on the DMF, and the limit of detection is as low as 0.01 μM. The proposed method based on AuNPs with the advantages of automation, low reagent cost, and small size could be further developed for a wide range of applications such as point-of-care testing of nucleic acid, antibody, proteins, and small molecules.
